# Supplementary material for: Prion shedding is reduced by chronic wasting disease vaccination
Source: PLoS Pathog. 2026 Apr 24;22(4):e1014166. doi: 10.1371/journal.ppat.1014166 (PMC13128116; doi:10.1371/journal.ppat.1014166)
Supplement: S1 Fig — The y-axis represents the optical density (OD) at 405 nm in ELISA, indicating the reactivity of post-immune sera from either Ddi (A) or Mmo (B) immunized mice against linear epitopes of deer PrP on the x-axis. The dashed horizontal line represents the cut-off, which is 3 times the average of preimmune sera. The data in panel (A) represents the average of Ddi sera from mice 1, 3, 4 and 6. The data in panel (B) represents the average of Mmo sera from mice 1, 2, 3 and 6. Data are presented as mean ± SD of results from four individual mice of each group. The amino acid sequences for linear epitopes are shown in S1 Table. (PDF) [file ppat.1014166.s001.pdf]

S1 Fig

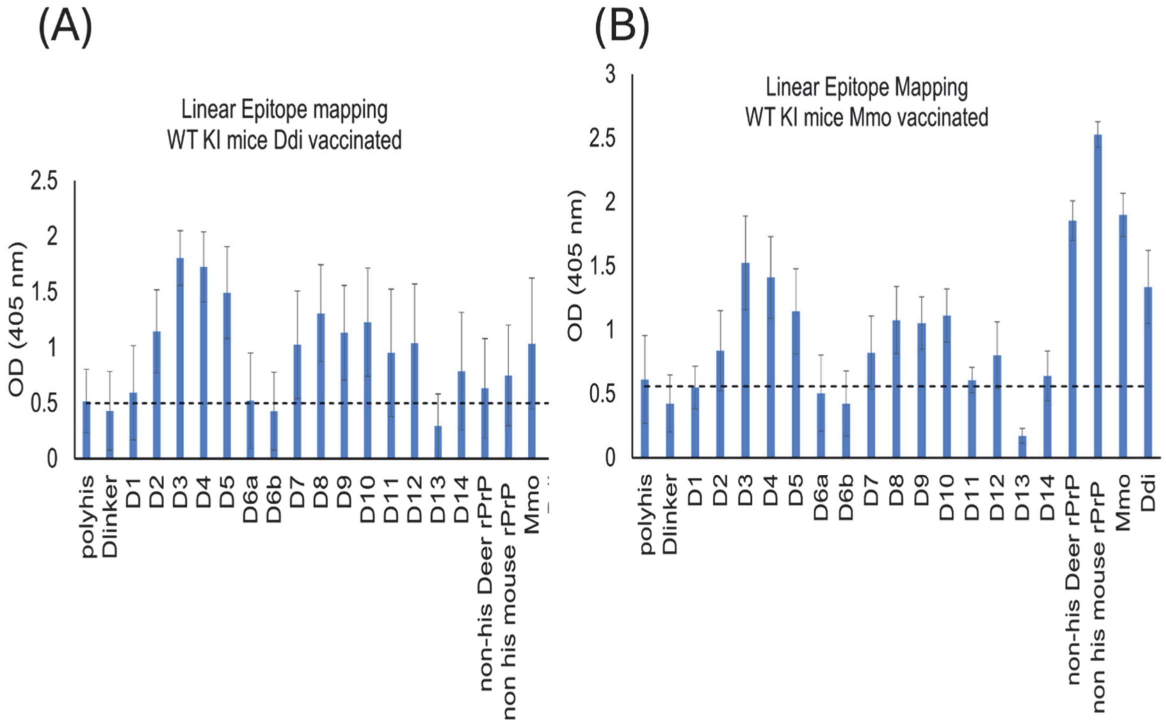

S1 Fig. Linear epitope mapping of sera from Ddi and Mmo vaccinated mice. The y-axis represents the optical density (OD) at 405 nm in ELISA, indicating the reactivity of post-immune sera from either Ddi **(A)** or Mmo **(B)** immunized mice against linear epitopes of deer PrP on the x-axis. The dashed horizontal line represents the cut-off, which is 3 times the average of pre-immune sera. The data in panel **(A)** represents the average of Ddi sera from mice 1, 3, 4 and 6. The data in panel **(B)** represents the average of Mmo sera from mice 1, 2, 3 and 6. Data are presented as mean ± SD of results from four individual mice of each group. The amino acid sequences for linear epitopes are shown in S1 Table.
