# Supplementary material for: Prion shedding is reduced by chronic wasting disease vaccination
Source: PLoS Pathog. 2026 Apr 24;22(4):e1014166. doi: 10.1371/journal.ppat.1014166 (PMC13128116; doi:10.1371/journal.ppat.1014166)
Supplement: S2 Fig — Fecal homogenates from individual mice were taken at 250 dpi and extracted using IOME followed by three rounds of PMCA reactions seeded with 10–1 dilution of 10% fecal homogenates and performed in duplicates. Positive control for PMCA was naïve feces spiked with mouse-adapted CWD and negative control was naïve feces. PMCA products were analyzed using RT-QuIC at 10–2 dilution. (A-C), representative RT-QuIC graphs showing the seeding activity in feces from vaccinated or CpG control mice. Samples were considered positive when 2 out of 4 wells crossed the threshold, which is defined as the average RFU of the negative control group plus five times its standard deviation. The y-axis represents the RFU, and the x-axis represents the time in hours (hr). (D) Chi square test, (E) time to threshold, (F) maximum of range, and (G) area under curve. Graphs were generated using GraphPad Prism (version 10). Statistical analysis was done using Chi-square test with **** p-value < 0.0001, or One-way ANOVA followed by a Tukey’s multiple comparison. ns means not significant. (PDF) [file ppat.1014166.s002.pdf]

S2 Fig

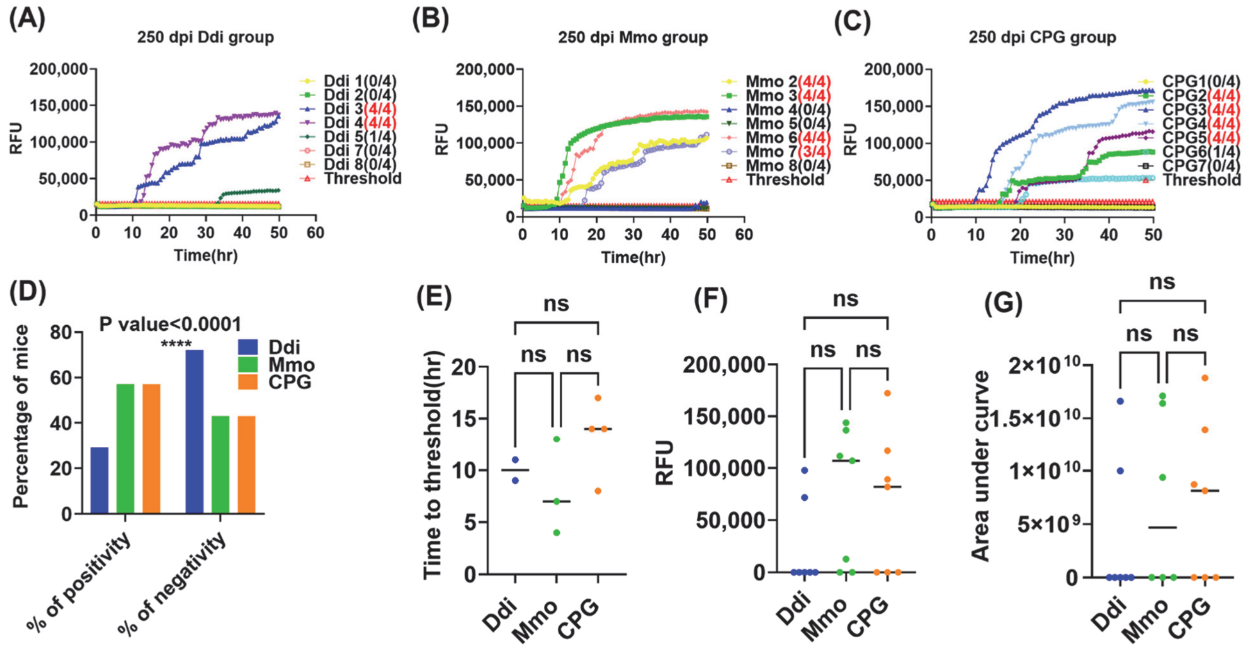


**S2 Fig. RT-QuIC data showing the seeding activity in feces from vaccinated and control mice at 250 dpi.** Fecal homogenates from individual mice were taken at 250 dpi and extracted using IOME followed by three rounds of PMCA reactions seeded with $10^{-1}$ dilution of 10% fecal homogenates and performed in duplicates. Positive control for PMCA was naïve feces spiked with mouse-adapted CWD and negative control was naïve feces. PMCA products were analyzed using RT-QuIC at $10^{-2}$ dilution. **(A-C),** representative RT-QuIC graphs showing the seeding activity in feces from vaccinated or CpG control mice. Samples were considered positive when 2 out of 4 wells crossed the threshold, which is defined as the average RFU of the negative control group plus five times its standard deviation. The y-axis represents the RFU, and the x-axis represents the time in hours (hr). **(D)** Chi square test, **(E)** time to threshold, **(F)** maximum of range, and **(G)** area under curve. Graphs were generated using GraphPad Prism (version 10). Statistical analysis was done using Chi-square test with **** p-value < 0.0001, or One-way ANOVA followed by a Tukey's multiple comparison. ns means not significant.
