# Supplementary material for: Prion shedding is reduced by chronic wasting disease vaccination
Source: PLoS Pathog. 2026 Apr 24;22(4):e1014166. doi: 10.1371/journal.ppat.1014166 (PMC13128116; doi:10.1371/journal.ppat.1014166)
Supplement: S3 Fig — Fecal homogenates from individual mice taken at 300 dpi were extracted and subjected to IPR analysis. IOME extraction was followed by three rounds of PMCA reactions seeded with 10–1 dilution of 10% fecal homogenates. Positive control for PMCA was naïve feces spiked with mouse-adapted CWD and negative control was naïve feces. All samples and controls were subjected to PMCA and products analyzed using RT-QuIC at 10–1 dilution. (A-C), representative RT-QuIC graphs showing the seeding activity in feces from vaccinated or control mice. Samples were considered positive when 2 out of 4 wells crossed the threshold, which is defined as the average RFU of the negative control group plus five times its standard deviation. The y-axis represents the RFU, and the x-axis represents the time in hours (hr). (D) Chi square test, (E) time to threshold, (F) maximum of range, and (G) area under curve. Graphs were generated using GraphPad Prism (version 10). Statistical analysis done using Chi-square test with **** p-value < 0.0001 or One-way ANOVA followed by a Tukey’s multiple comparison for time to threshold (E): Ddi vs. Mmo ** p-value = 0.0043, Ddi vs. CpG ** p-value = 0.0037; for maximum of range (F): Ddi vs. Mmo * p-value = 0.0498, Ddi vs. CpG * p-value = 0.0149. ns: not significant; and for area under curve (G): Ddi vs. Mmo * p-value = 0.0225, Ddi vs. CpG * p-value = 0. 0120. (PDF) [file ppat.1014166.s003.pdf]

S3 Fig

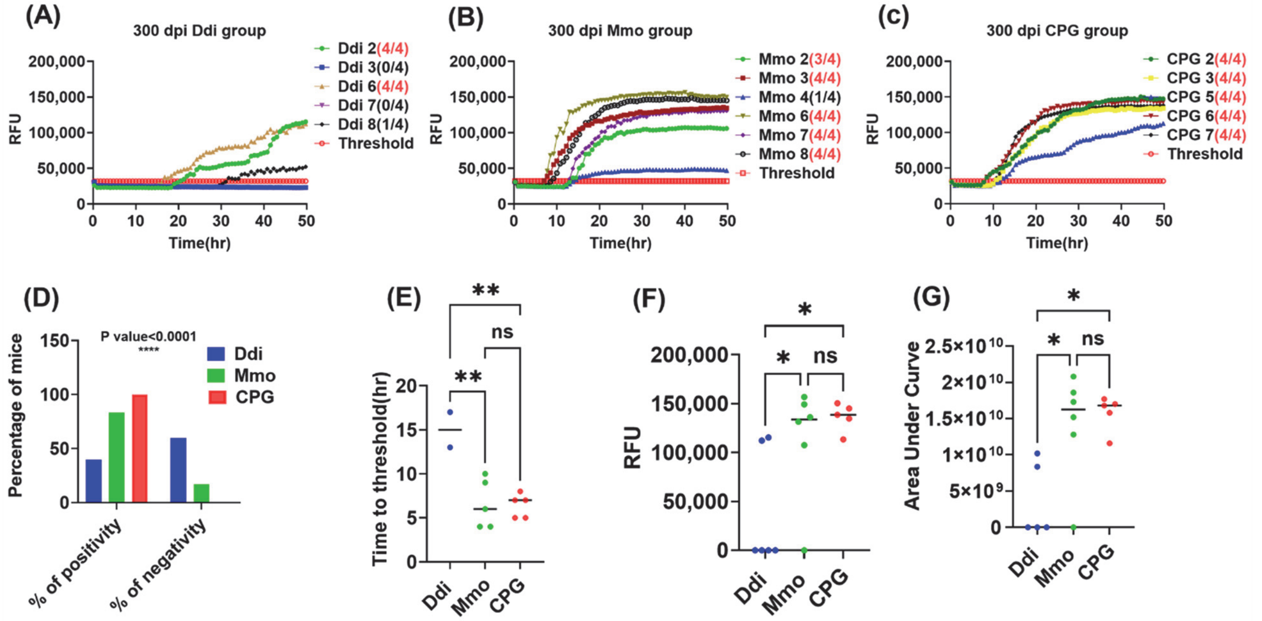

S3 Fig. Seeding activity in feces from vaccinated and control mice at 300 dpi. Fecal homogenates from individual mice taken at 300 dpi were extracted and subjected to IPR analysis. IOME extraction was followed by three rounds of PMCA reactions seeded with 10^{-1} dilution of 10% fecal homogenates. Positive control for PMCA was naïve feces spiked with mouse-adapted CWD and negative control was naïve feces. All samples and controls were subjected to PMCA and products analyzed using RT-QuIC at 10^{-1} dilution. **(A-C),** representative RT-QuIC graphs showing the seeding activity in feces from vaccinated or control mice. Samples were considered positive when 2 out of 4 wells crossed the threshold, which is defined as the average RFU of the negative control group plus five times its standard deviation. The y-axis represents the RFU, and the x-axis represents the time in hours (hr). **(D)** Chi square test, **(E)** time to threshold, **(F)** maximum of range, and **(G)** area under curve. Graphs were generated using GraphPad Prism (version 10). Statistical analysis done using Chi-square test with **** p-value < 0.0001 or One-way ANOVA followed by a Tukey's multiple comparison for time to threshold **(E)**: Ddi vs. Mmo ** p-value = 0.0043, Ddi vs. CpG ** p-value = 0.0037; for maximum of range **(F)**: Ddi vs. Mmo * p-value = 0.0498, Ddi vs. CpG * p-value = 0.0149. ns: not significant; and for area under curve **(G)**: Ddi vs. Mmo * p-value = 0.0225, Ddi vs. CpG * p-value = 0. 0120.
