# Supplementary material for: Prion shedding is reduced by chronic wasting disease vaccination
Source: PLoS Pathog. 2026 Apr 24;22(4):e1014166. doi: 10.1371/journal.ppat.1014166 (PMC13128116; doi:10.1371/journal.ppat.1014166)
Supplement: S4 Fig — Fecal samples taken from individual mice at 350 dpi were extracted using IOME and subjected to three rounds of PMCA. Positive control for the PMCA was naïve feces spiked with mouse-adapted CWD and negative control was naïve feces. All samples and controls were subjected to PMCA and products analyzed using RT-QuIC at 10–5 dilution. (A-C), representative RT-QuIC graphs showing the seeding activity in feces from vaccinated or control mice. Samples were considered positive when 2 out of 4 wells crossed the threshold, which is defined as the average RFU of the negative control group plus five times its standard deviation. The y-axis represents the RFU, and the x-axis represents the time in hours (hr). (D) Chi square test, (E) time to threshold, (F) maximum of range, and (G) area under curve. Graphs were generated using GraphPad Prism (version 10). Statistical analysis was done using Chi-square test with **** p-value < 0.0001, or Two-way ANOVA followed by a Tukey’s multiple comparison for area under curve (G): Ddi vs CpG * p-value = 0.0453 and Mmo vs CpG ** p-value = 0.0052; and for maximum of range (F): * p-value = 0.0308. (PDF) [file ppat.1014166.s004.pdf]

S4 Fig

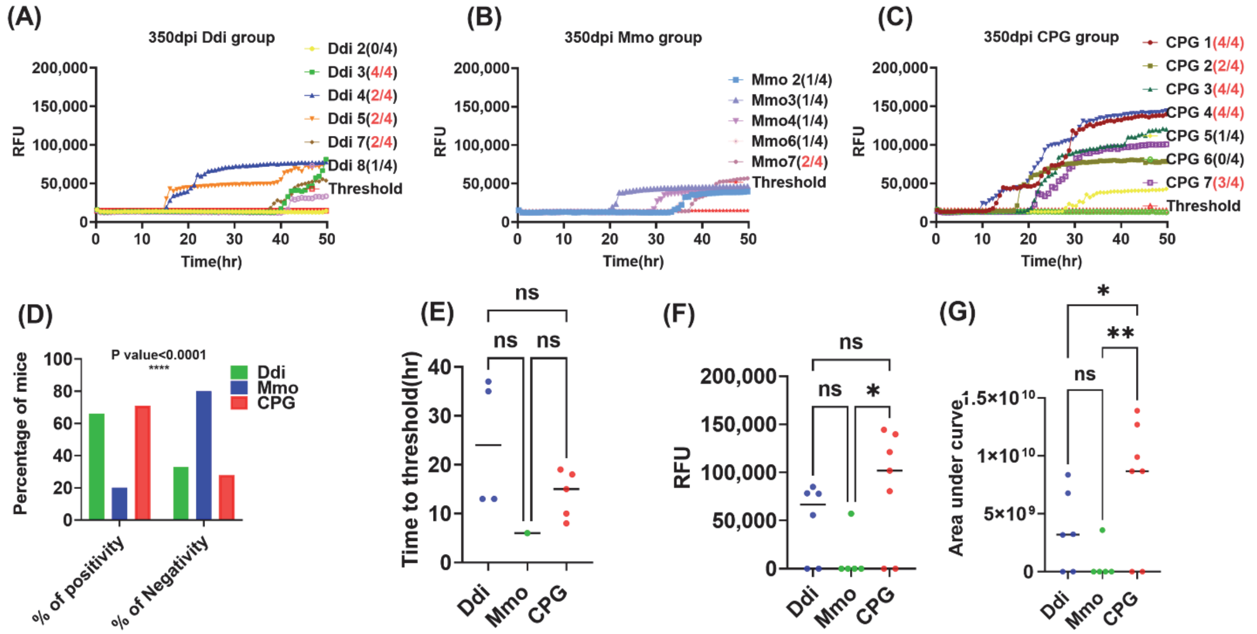

S4 Fig. Seeding activity in feces from vaccinated and control mice at 350 dpi. Fecal samples taken from individual mice at 350 dpi were extracted using IOME and subjected to three rounds of PMCA. Positive control for the PMCA was naïve feces spiked with mouse-adapted CWD and negative control was naïve feces. All samples and controls were subjected to PMCA and products analyzed using RT-QuIC at 10^{-5} dilution. **(A-C),** representative RT-QuIC graphs showing the seeding activity in feces from vaccinated or control mice. Samples were considered positive when 2 out of 4 wells crossed the threshold, which is defined as the average RFU of the negative control group plus five times its standard deviation. The y-axis represents the RFU, and the x-axis represents the time in hours (hr). **(D)** Chi square test, **(E)** time to threshold, **(F)** maximum of range, and **(G)** area under curve. Graphs were generated using GraphPad Prism (version 10). Statistical analysis was done using Chi-square test with **** p-value < 0.0001, or Two-way ANOVA followed by a Tukey's multiple comparison for area under curve **(G):** Ddi vs CpG * p-value = 0.0453 and Mmo vs CpG ** p-value = 0.0052; and for maximum of range **(F):** * p-value =0.0308.
