# Supplementary material for: Prion shedding is reduced by chronic wasting disease vaccination
Source: PLoS Pathog. 2026 Apr 24;22(4):e1014166. doi: 10.1371/journal.ppat.1014166 (PMC13128116; doi:10.1371/journal.ppat.1014166)
Supplement: S5 Fig — The graphs represent RT-QuIC results of serial dilutions (10–1 to 10–6) for 150 dpi (A) and 250 dpi (B) pooled urine samples from all 3 groups after three rounds of PMCA using mouse rPrP substrate. Fluorescence signals were measured every 15 min. The x-axis represents the reaction time (hour), the y-axis the relative fluorescence units (RFU). The threshold was based on the average fluorescence values of the negative control + 5 × SD used in every assay. Each curve represents an average of 4 technical replicates. (PDF) [file ppat.1014166.s005.pdf]

S5 Fig

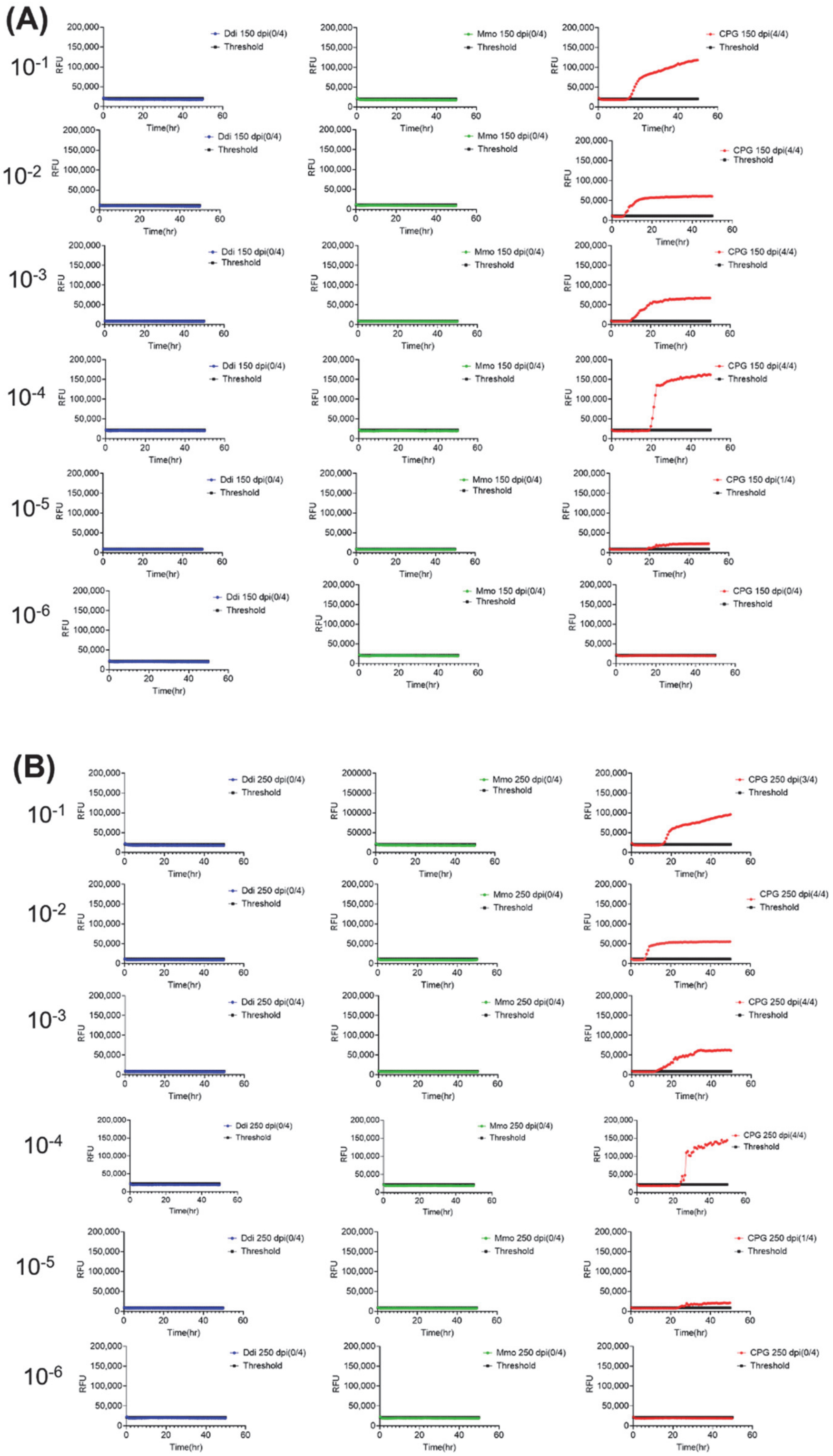

S5 Fig. RT-QuIC prion seeding activity in third round PMCA products for urine of mice vaccinated with Ddi or Mmo and control CpG group. The graphs represent RT-QuIC results of serial dilutions (10^{-1} to 10^{-6}) for 150 dpi **(A)** and 250 dpi **(B)** pooled urine samples from all 3

groups after three rounds of PMCA using mouse rPrP substrate. Fluorescence signals were measured every 15 min. The *x*-axis represents the reaction time (hour), the *y*-axis the relative fluorescence units (RFU). The threshold was based on the average fluorescence values of the negative control + 5 × SD used in every assay. Each curve represents an average of 4 technical replicates.
