# Supplementary material for: Prion shedding is reduced by chronic wasting disease vaccination
Source: PLoS Pathog. 2026 Apr 24;22(4):e1014166. doi: 10.1371/journal.ppat.1014166 (PMC13128116; doi:10.1371/journal.ppat.1014166)
Supplement: S6 Fig — The graphs represent results of serially diluted RT-QuIC reactions (10-1 to 10-–6) for urine samples taken at 450 dpi after three rounds of PMCA. Fluorescence signals were measured every 15 min. The x-axis represents the reaction time (hours), the y-axis represents the relative fluorescence units (RFU). The threshold was based on the average fluorescence values of the negative control + 5 × SD used in every assay. Each curve represents the average of 4 technical replicates. (PDF) [file ppat.1014166.s006.pdf]

S6 Fig

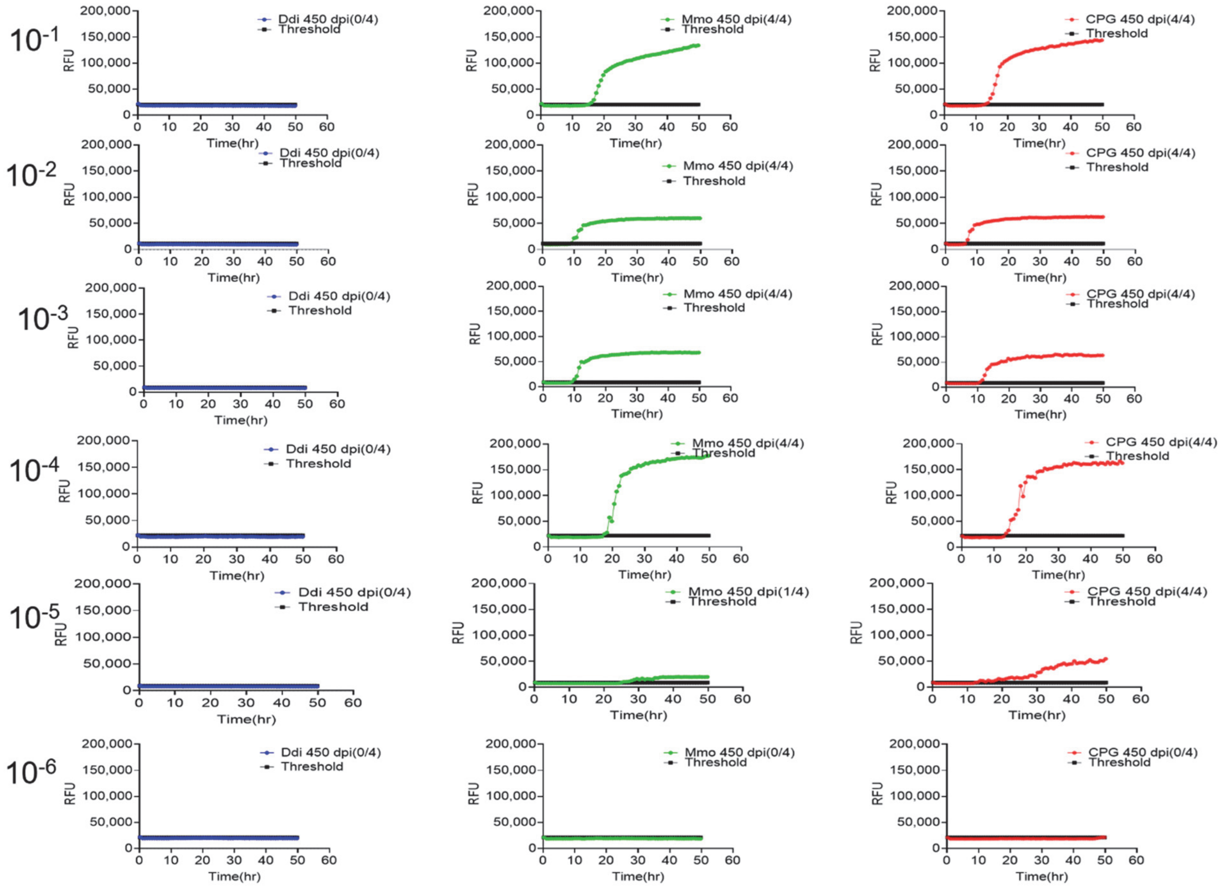

S6 Fig. RT-QuIC data showing the CWD prion seeding activity in third round PMCA products for urine of mice at 450 dpi. The graphs represent results of serially diluted RT-QuIC reactions (10^{-1} to 10^{-6}) for urine samples taken at 450 dpi after three rounds of PMCA. Fluorescence signals were measured every 15 min. The x-axis represents the reaction time (hours), the y-axis represents the relative fluorescence units (RFU). The threshold was based on the average fluorescence values of the negative control + 5 × SD used in every assay. Each curve represents the average of 4 technical replicates.
