# Supplementary material for: Prion shedding is reduced by chronic wasting disease vaccination
Source: PLoS Pathog. 2026 Apr 24;22(4):e1014166. doi: 10.1371/journal.ppat.1014166 (PMC13128116; doi:10.1371/journal.ppat.1014166)
Supplement: S7 Fig — Fluorescence signals were measured every 15 min. The x-axis represents the reaction time (hours), the y-axis represents the relative fluorescence units (RFU). The threshold was based on the average fluorescence values of the negative control + 5 × SD used in every assay. Each curve represents an average of 4 technical replicates. (PDF) [file ppat.1014166.s007.pdf]

S7 Fig

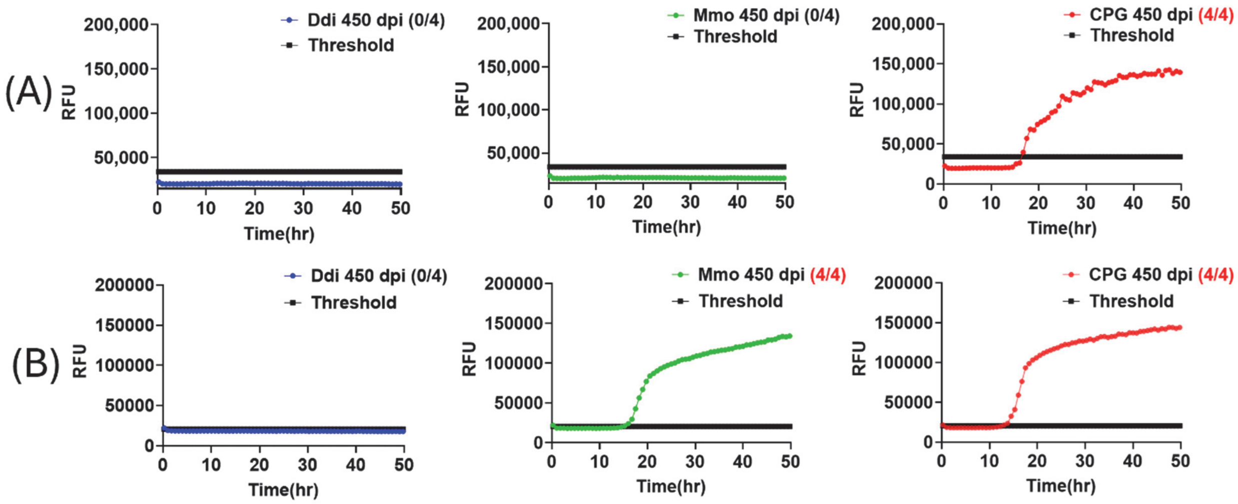

S7 Fig. RT-QuIC data showing the seeding activity in 450 dpi pooled urine samples extracted with IOME (A) or IOME followed by three rounds of PMCA (B). Fluorescence signals were measured every 15 min. The *x*-axis represents the reaction time (hours), the *y*-axis represents the relative fluorescence units (RFU). The threshold was based on the average fluorescence values of the negative control + 5 × SD used in every assay. Each curve represents an average of 4 technical replicates.
