# Supplementary material for: Prion shedding is reduced by chronic wasting disease vaccination
Source: PLoS Pathog. 2026 Apr 24;22(4):e1014166. doi: 10.1371/journal.ppat.1014166 (PMC13128116; doi:10.1371/journal.ppat.1014166)
Supplement: S8 Fig — ELISArepresenting the reactivity of (A) feces from Ddi vaccinated mice (300 dpi) diluted 1:50 (w/v), or(B) 1:10 diluted urine, was tested using Ddi antigen to coat the plate. (C) represents the reactivityof feces from Mmo-vaccinated mice (300 dpi) diluted 1:50 (w/v) and (D) represents 1:10 dilutedurine against Mmo as antigen used to coat the plate. For all panels goat anti-mouse detecting IgG, IgA and IgM was used as secondary antibody. Urine was pooled per cage, so only two samples per group were analyzed. (PDF) [file ppat.1014166.s008.pdf]

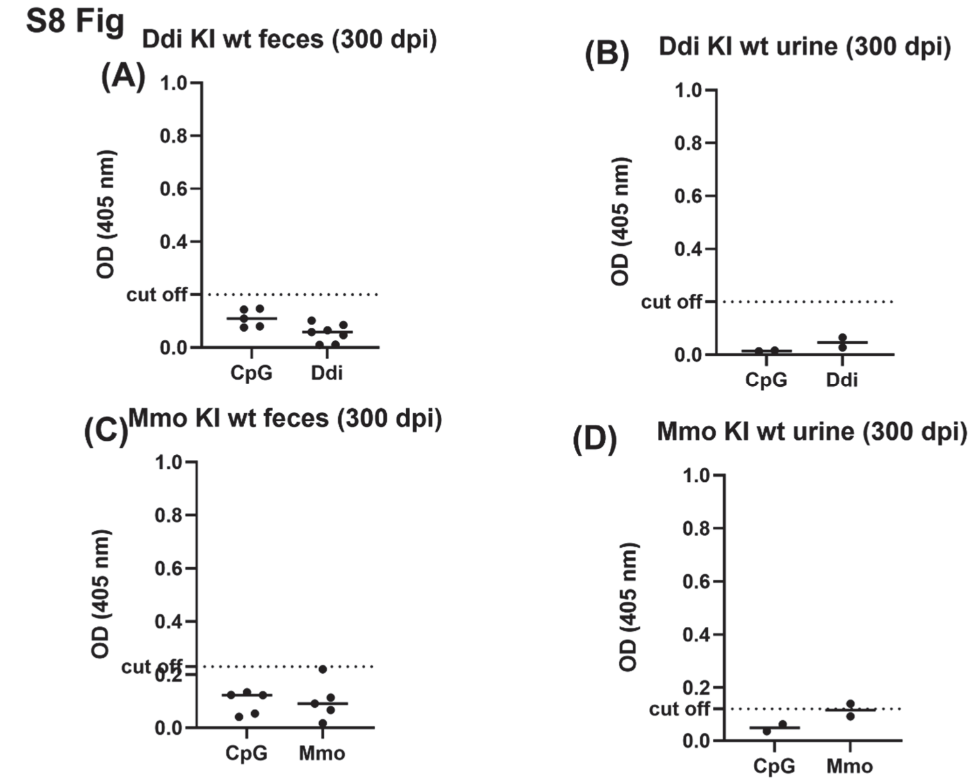

S8 Fig. No anti-PrP antibodies were detected in feces and urine of vaccinated mice. ELISA representing the reactivity of **(A)** feces from Ddi vaccinated mice (300 dpi) diluted 1:50 (w/v), or **(B)** 1:10 diluted urine, was tested using Ddi antigen to coat the plate. **(C)** represents the reactivity of feces from Mmo-vaccinated mice (300 dpi) diluted 1:50 (w/v) and **(D)** represents 1:10 diluted urine against Mmo as antigen used to coat the plate. For all panels goat anti-mouse detecting IgG, IgA and IgM was used as secondary antibody. Urine was pooled per cage, so only two samples per group were analyzed.
