# Supplementary material for: Prion shedding is reduced by chronic wasting disease vaccination
Source: PLoS Pathog. 2026 Apr 24;22(4):e1014166. doi: 10.1371/journal.ppat.1014166 (PMC13128116; doi:10.1371/journal.ppat.1014166)
Supplement: S1 Table — (PDF) [file ppat.1014166.s009.pdf]

S1 Table. Sequence of deer PrP peptides used in epitope mapping.

Epitope	Sequence
Polyhis-tag	MRG SHH HHH HGS CKK RPK PG
linker	QRGASAGA IGG AKK RPK P
D.1	KKRPKPGGGWNTGGSRYPGQG
D.2	YPGQGSPGGNRYPPQGGGGWG
D.3	GGGGWGQPHGGGWGQPHGGGW
D.4	HGGGWGQPHGGGWGQPHGGG
D.5	PHGGGGWGQGGTHSQWNKPS
D.6a	WNKPSKPKTNMKHVAGAAAA
D.6b	WNKPSKPKTNMKHMAGAAAA
D.7	GAAAAGAVVGGLGGYMLGSA
D.8	MLGSAMSRPLIHFGNDYEDR
D.9	DYEDRYYRENMYRYPNQVYY
D.10	NQVYYRPVDQYNNQNTFVHD
D.11	NTFVHDCVNITVKQHTVTTTT
D.12	VTTTTKGENFTETDIKMMER
D.13	KMMERVVEQMCITQYQRESQ
D.14	QRESQAYYQRGAS
