# Supplementary material for: Prion shedding is reduced by chronic wasting disease vaccination
Source: PLoS Pathog. 2026 Apr 24;22(4):e1014166. doi: 10.1371/journal.ppat.1014166 (PMC13128116; doi:10.1371/journal.ppat.1014166)
Supplement: S2 Table — (PDF) [file ppat.1014166.s010.pdf]

S2 Table. Number of mice per group for pooled urine analysis.

	150 dpi	250 dpi	450dpi
Ddi	7 mice (all except #6)	7 mice (all except #6)	3mice (#2, #3, #4)
Mmo	8 mice (#1 - #8)	7 mice (#2 - #8)	2mice (#2, #4)
CPG	7 mice (#1 - #7)	7 mice (#1 - #7)	1 mouse (#6)
