# Supplementary material for: Prion shedding is reduced by chronic wasting disease vaccination
Source: PLoS Pathog. 2026 Apr 24;22(4):e1014166. doi: 10.1371/journal.ppat.1014166 (PMC13128116; doi:10.1371/journal.ppat.1014166)
Supplement: S3 Table — (PDF) [file ppat.1014166.s011.pdf]

S3 Table. PrP^C antibody titer and positive replicates in CWD seeding activity in feces at different time points.

ID	200 dpi	250 dpi	300dpi	350dpi	fold dilution
Ddi 1	0	0	ND*	ND*	10,000
Ddi 2	0	0	100	0	30,000
Ddi 3	50	100	0	100	ND#
Ddi 4	50	100	ND	50	30,000
Ddi 5	0	0	ND	50	30,000
Ddi 6	100	ND	100	ND	40,000
Ddi 7	0	0	0	50	50,000
Ddi 8	100	0	0	0	50,000
Mmo 1	50	ND*	ND*	ND*	10,000
Mmo 2	0	100	75	0	30,000
Mmo 3	100	100	100	0	30,000
Mmo 4	100	0	0	0	30,000
Mmo 5	75	0	ND	ND	30,000
Mmo 6	100	100	100	0	30,000
Mmo 7	75	75	100	50	30,000
Mmo 8	75	0	100	ND	5,000
CPG 1	100	0	ND	100	
CPG 2	50	50	100	50	
CPG 3	50	100	100	100	
CPG 4	100	100	ND	100	
CPG 5	100	100	100	0	
CPG 6	100	0	100	0	
CPG 7	100	ND	100	75	
CPG 8	ND*	ND*	ND*	ND*	

ND*: mouse euthanized before this time point, ND#: not enough sera collected and ND: feces could not be collected from this mouse. The scale ranges from 0 (all replicates were negative) to 100 (all replicates were positive).
