# Supplementary material for: Prion shedding is reduced by chronic wasting disease vaccination
Source: PLoS Pathog. 2026 Apr 24;22(4):e1014166. doi: 10.1371/journal.ppat.1014166 (PMC13128116; doi:10.1371/journal.ppat.1014166)
Supplement: Attachment — Submitted filename: rebuttal letter PPATHOGENS-D-25-03279.pdf [file ppat.1014166.s013.pdf]

We thank the reviewers for their valuable and highly constructive comments. We have addressed all of them and accordingly modified our manuscript. With this careful modification, we hope that the manuscript is now improved and appropriate for publication.

Our responses are provided below in full detail. Line numbering refers to current version that indicates changes (marked-up copy; in track mode).

Additional Editor Comments:
Please be mindful of the comments of the three reviews and pay special attention to Review 2, major concern 1 about how the presence of anti-PrP antibodies in feces and urine may affect PMCA detection and Reviewer 3 on discussing the differences between murine and cervid GI tracks.

Both comments were appropriately addressed. The presence of anti-PrP antibodies in feces and urine was directly tested in Elisa and negative results are presented as a **new Supplementary Figure 8**. We also tested in spiking experiments whether the presence of antibodies would interfere with the IPR readout method, results provided as a **new Figure 6**. Outcome is they do not interfere. We have discussed the differences between murine and cervid GI tracks now in the Discussion.

Part II – Major Issues: Key Experiments Required for Acceptance

Reviewer #2: Major concern 1:
The authors should address whether residual antibodies in feces and urine samples, even at minimal levels, could interfere with the initial PMCA amplification step. Since direct detection of prions is not possible in these samples, the presence of vaccine-induced antibodies (either free or already bound to PrPres seeds) could affect amplification efficiency and lead to misinterpretation of results. This concern is particularly relevant because the differences observed between vaccinated and control groups could potentially reflect assay interference rather than actual differences in prion shedding.
The authors should discuss this possibility and, if feasible, provide experimental evidence that antibodies do not interfere with the IPR technique. This could include spiking experiments with known amounts of prions in the presence of immune sera at concentrations expected in excreta.

We thank the referee for bringing up this very important point.
We have now performed the suggested spiking experiments and found that anti-PrP^C antibodies do not interfere with the IPR readout. Naïve mouse feces were treated with pre-immune or post-immune sera of various KI mice described in this study, followed by spiking with CWD-positive brain homogenate. IOME enrichment was applied to all samples, followed by three rounds of PMCA in duplicate, and RT-QuIC in quadruplicate. Importantly, naïve feces were also treated with defined monoclonal and polyclonal anti-PrP sera (mAb 4H11 or pAb 142; both at 1:500 dilution) before spiking and analysis. This setting reflects a situation with very high presence of antibodies.
We also tested for the presence of anti-PrP antibodies in fecal samples and urine. Feces from individual mice were diluted 1:50, pooled urine 1:10, and tested in Elisa for reactivity against

the Ddi or Mmo antigen on the plate. A goat anti-mouse secondary antibody (1:3,000) was used that detects IgG, IgA and IgM. We could not detect anti-PrP antibodies in these samples. These data are shown now as **new Fig. 6 and additional SFig. 8**. Outcomes from these experiments have strongly improved the conclusions of our manuscript, excluding that the differences observed between vaccinated and control groups could potentially reflect assay interference rather than actual differences in prion shedding (Discussion lines 430-435).

Major concern 2

Throughout the manuscript, the authors make statements that could be misinterpreted by readers regarding prion concentrations and fold-reductions (e.g., "5-log reduction," "high prion concentration," "10e-5 dilutions"). The read-out method combines purification/amplification by PMCA with re-amplification by RT-QuIC. This methodology, used because samples contain minuscule amounts of PrPres (seeds), means that extremely small differences in initial PrPres quantity are enormously amplified. Therefore, statements about "log reductions" could be misinterpreted as referring to direct measurements of seed quantities, which is not accurate.

Specific examples to revise:

- *Lines referencing "a 5-log reduction of shedding in Ddi-vaccinated mice"*
- *"We observed high prion concentration in both feces and urine samples"*
- *"Pooled urine samples from the CpG control group showed seeding activity until the 10e-5 dilution at 450 dpi"*
- *"...whereas 4 serial dilutions of urine tested positive for CpG-only treated mice"*

The authors must revise these statements throughout the manuscript to clarify that these refer to seeding activity in amplified products, not direct prion concentrations. They should avoid language that could mislead readers about the actual quantities of prions in the original samples.

We agree that these statements might have been misleading and have accordingly adjusted our conclusions and all the specific examples mentioned above by the referee. We also added to the text that we did not perform direct measurements of PrP^{Sc} in urine and feces and that all values we present and discuss are the result of prior seed amplification (PMCA) and subsequent prion conversion (RT-QuIC), both highly sensitive assays that likely enlarge very small differences in initial PrP^{Sc} quantity upon amplification (lines 411-414 Discussion: “Of note, our IPR readout method combines purification and amplification by PMCA with a re-amplification by RT-QuIC. This is necessary because rodent urine and feces contain only very small amounts of PrP^{Sc} and could mean that very small differences in initial PrP^{Sc} quantities could be enormously amplified in a non-linear way.”).

Major concern 3

The authors should provide better justification and discussion of the necessity of using a coupled method involving two different amplification techniques (PMCA + RT-QuIC) and how this might influence interpretation of observed differences. The use of serial PMCA (3

rounds) followed by RT-QuIC introduces variability that is well-documented for PMCA. The discussion should address:

1. *Why this coupled approach was necessary for this particular study*
2. *How the known variability of serial PMCA might affect the interpretation of differences between groups*
3. *Whether the differences observed could be influenced by the amplification process itself rather than solely reflecting differences in original prion content*

We thank the reviewer for this important comment. We have extensively evaluated the IPR assay for mouse samples, comparing the combined assay with direct PMCA amplification alone or RT-QuIC alone. In fact, we initially wanted to publish this very detailed evaluation work as a stand-alone manuscript. Major outcome was that IPR increases the specificity of the readout while keeping the sensitivity very high, or even higher (see also **SFig. 7**). Specificity is known in the prion field to be very problematic when using urine and feces as a test matrix. Feces is a complex matrix that contains proteases, microbial proteins and auto-fluorescent compounds. When we started analysing prion shedding in feces, we tried various approaches including prion enrichment using phospho-tungstic acid, or iron oxide magnetic extraction followed by RT-QuIC alone (no PMCA). We noticed positive seeding activity in many naïve samples as shown in the following figure.

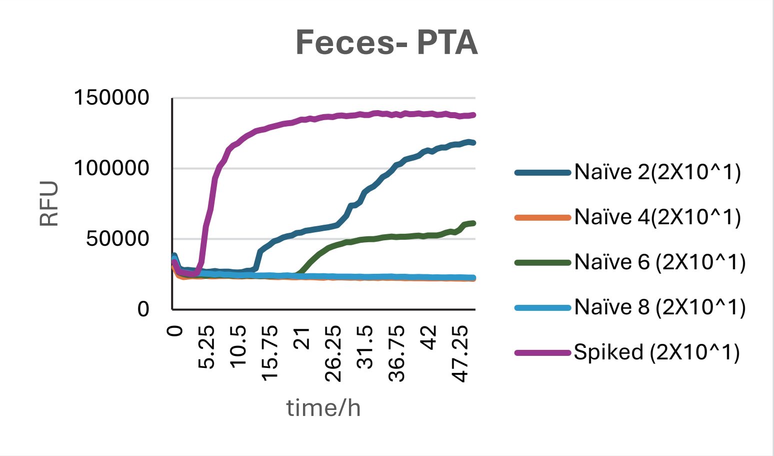

Rebuttal Fig. 1: RT-QuIC assay for four different naïve fecal samples and a spiked sample serving as positive control (shown in purple color). All samples were homogenized in feces homogenization buffer to reach 10% (w/v) followed by prion enrichment using Na-phospho-tungstic acid. As shown in the figure two out of four naïve samples turned strongly positive.

In addition, in the cervid system, Denkers et al. (2024) reported many false positives after prion enrichment using iron oxide magnetic extraction when followed by RT-QuIC, as shown in the figure below. To get rid of the false-positive background, they used 4 rounds of PMCA after IOME (we only did 3 rounds). The PMCA products were analysed using RT-QuIC as read-out. In their hands, combination of PMCA and RT-QuIC eliminated false positives in fecal samples and led to enhanced and more reliable and robust prion seeding activity detection in feces. This was also the outcome of our own validation study when using mouse samples.

Our very extensive own testing did not indicate that the potential variability of serial PMCA might affect the interpretation of differences between groups or that the differences observed could be influenced by the amplification process itself rather than solely reflecting differences in original prion content.

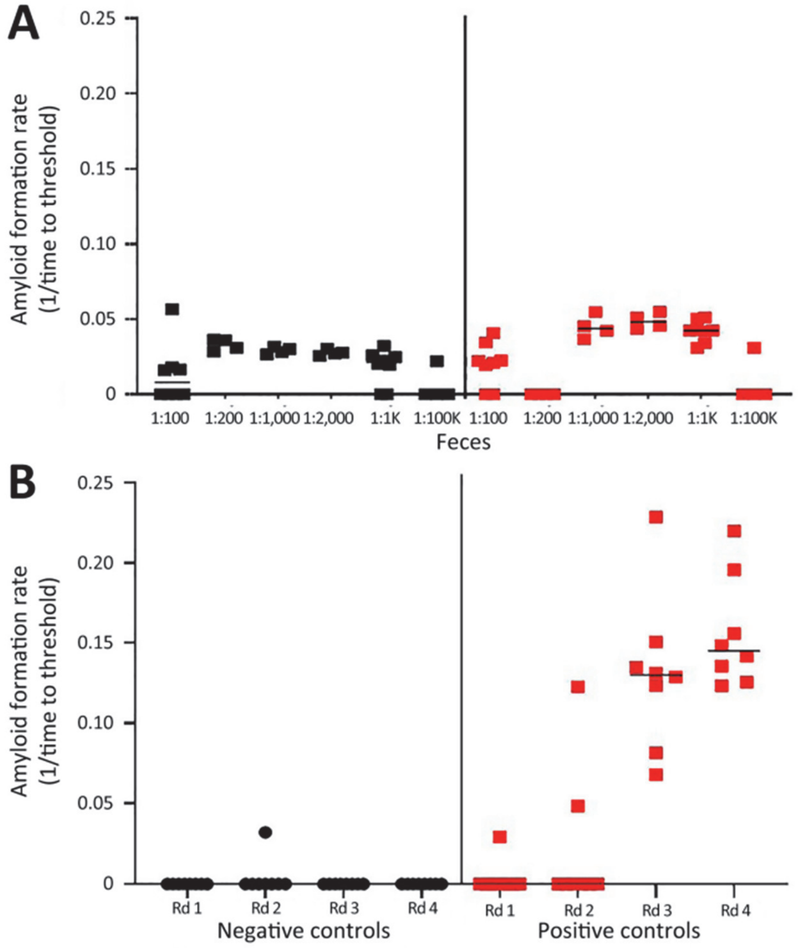

Figure 1. Amyloid formation rates in fecal samples from white-tailed deer with chronic wasting disease in longitudinal study of temporal characterization of prion shedding in secreta, United States. A) Amyloid formation rates measured by using iron oxide bead capture and subsequent real-time quaking-induced conversion. Rates were measured for serial dilutions of fecal samples. B) Amyloid formation rates measured by using iron oxide bead capture, 4 rounds of serial protein misfolding amplification, and subsequent real-time quaking-induced conversion. Rates were measured for 1:100 dilutions of fecal samples from each round of amplification. Black indicates prion-negative feces; red indicates prion-positive feces. Horizontal lines in each grouping indicate median values. Rd, round.

Denkers et al., 2024

Reviewer #3:

1. *Does PrP decrease on peripheral cells (FDCs, B cells, eg) after vaccination, either transiently or during disease course, that might contribute to less prion replication and shedding? If so, any adverse side effects from decreased PrP expression?*

We thank the referee for this interesting comment. In previous cell culture experiments we could observe that continued application of rather high titers of anti-PrP antibodies could decrease surface expression of PrP (described in Gilch et al., 2003 and Abdelaziz et al., 2017). This effect was only observed when using full IgG, not for Fab fragments, and likely represents cell surface clustering events. To see this effect, it needs high antibody concentrations and repeated treatments with fresh antibody material. Immunofluorescence data indicated that the effect is largely transient and fades over time, as new PrP^C is constantly produced by the cells and internalized antibodies/PrP^C-antibody complexes are also degraded (removing the anti-PrP antibodies).

We have no experimental evidence that such reduction of PrP^C also occurs *in vivo*, in vaccinated mice (for example when comparing -PK samples in immunoblot, from brain,

spinal cord and spleen homogenates). We very carefully analyzed our vaccinated mice for potential side effects by weekly clinical assessment and weekly weighing. Pathology and histopathology analysis of harvested mice did not show abnormalities in spleen, kidney and brain, although some lymph node enlargement is found sometimes. Similarly, potential adjuvant toxicity was tested and no indication for e.g. weight loss, hepatotoxicity, splenomegaly or encephalitis was obtained. Most importantly, we have very robust side effect data from an ongoing vaccination study in white-tailed deer (WTD), where deer were immunized and boostered with Ddi as used here (systemic vaccination). Extensive blood work was done, testing for potential side effects via blood parameters, CBC and flow cytometry of immune cell profiles: both CWD-challenged and non-challenged deer are not being impacted with any negative blood parameters. This is a very promising and important outcome that widely recapitulates what we have seen so far in mice, transgenic mice, KO mice, reindeer, WTD and elk studies. We have added statements to the text (lines 295-299 in Discussion).

Part III – Minor Issues: Editorial and Data Presentation Modifications

Reviewer #1: *A key conclusion from the study is that vaccination delays neuro-invasion. While this conclusion is generally supported by the data, the graphs presented in Figures 1g, h, and i may not adequately illustrate this difference. The authors should consider employing alternative graph types, such as bar graphs, to more clearly depict the distinctions between vaccinated and unvaccinated subjects.*

We agree and have replaced the current graphs with bar graphs (Fig. 1 G-I).

There appear to be considerable discrepancies among individual vaccinated animals. For instance, the results from two Ddi cages in Figure 3b show significant variation. The authors should provide an explanation for this variability.

It was expected that prion shedding in general differs from mouse to mouse and also from time point to time point within an individual mouse, and that this might be different between feces and urine. This is known from cervid studies and was mentioned in the Discussion (references 11, 41, 43, 60 and 64). We expected this for the CpG control group and both vaccine groups, although with a trend of seeing less shedding in vaccinated mice.
In a pooling situation (4 mice in a cage) as shown in Fig. 3, we can have a mixture of no, low and high shedders, potentially outbalancing vaccine effects. To address this problem, we decided to perform the analysis from samples from individual mice (Fig. 4). This was possible for fecal samples, but not for urine. Still, there is individual variability, within and between animals and treatment groups.

Figure legends should be more informative, including details such as sample size (n). For example, Figures 2a and 2c lack explanations for the numbers displayed along the X-axis. Are these numbers representative of individual animals? Additionally, why are there no error bars?

We have added in the legends that sera were isolated from individual mice, numbered 1-8. Error bars for Elisa readings were added.

Figure 4d presents a chi-square test, yet there is no indication of statistical differences.

Statistical significance is added now to the figure.

The description in Figure 4e should include an explanation regarding the absence of statistical significance.

Done as suggested.

The discussion section could be shortened.

We have shortened the Discussion section. Lines 325-335, 339-343, 373-376 and 428-430 were removed (numbering refers to current version that indicates changes; marked-up copy in track mode).

Reviewer #2:
Minor issue 1
It would have been helpful if the text lines had been numbered to facilitate the review process.

We apologize, was done now.

Minor issue 2
In the introduction, it would be helpful to inform readers whether there is a differential shedding mechanism that might be influenced by the strain type used or the model type employed. In the discussion, the authors mention a cervid PrP expression model that does not exhibit shedding. This is important for clarifying that the model used, although a knock-in mouse whose physiology differs greatly from deer, might have shedding behavior similar to cervids. Please comment on this in the introduction to provide appropriate context.

We have included a short paragraph in the Introduction to address this (lines 97-100). The difference between transgenic and KI mice is also described in the Discussion (lines 317-323).

Minor issue 3
Reference 37 does not appear to demonstrate shedding in urine. Please verify whether an additional reference should be added here, such as reference 39 which is cited in the discussion regarding urine shedding.
This same comment applies to the statement in the first results section: "We used gene-targeted KI mice where mouse PrP was replaced with cervid PrP, which recapitulate the pathogenesis of CWD, including shedding of CWD prions into feces and urine."

We added previous reference 59 (now 38) from the Telling lab that describes co-housing transmission in their KI mice as a second reference for this statement. Reference 37 only describes CWD shedding in feces. This manuscript will be the first to describe CWD prion shedding in urine in a cervidized mouse model. There is plenty of literature for cervids, as mentioned in our manuscript.

Minor issue 4
In the results section, the authors state: "although a statistical analysis is not possible." Please clarify why statistical analysis was not possible in this context.

We have removed this statement. Only 5 mice in total reached terminal prion disease, many others were euthanized before reaching clinical endpoints, mostly due to intercurrent disease (one in CpG, two each in Ddi and Mmo groups). These low numbers do not allow to perform a statistical analysis.

Minor issue 5
The discussion section is excessively long and contains portions that repeat descriptions of results already presented. Please reduce the length of the discussion by eliminating redundant descriptions and focusing on interpretation and implications of the findings.

We agree and have therefore shortened the Discussion. Lines 325-335, 339-343, 373-376 and 428-430 were removed (numbering refers to current version that indicates changes, marked-up copy in track mode).

Minor issue 6
The statement "to correlate to ELISA titers to some extent" in the discussion does not accurately reflect what is shown in Supplementary Table 3. The table does not support a clear correlation. Please revise this statement to more accurately reflect the data presented.

We agree and have therefore deleted these statements.

Minor issue 7
Please discuss the changing percentages of shedding over time shown in Table 1. The variation in % shedding across timepoints (200, 250, 300, 350 dpi) within groups deserves interpretation.

Such variability was expected. This is known from cervid studies and is now better explained in the Discussion (references 11, 41, 43, 60 and 64). See also response to Referee 1, minor issue 01.
The variation in shedding over time has been previously reported in various publications. For example, in white- tailed deer, Denkers et al reported that shedding of CWD in feces reached 100% of the tested samples at 21 months post infection (mpi), then declined to 75% at 24 mpi and 66% at 27 mpi and reverted to 100 % at 29 mpi. Therefore, intermitted shedding has been observed in the natural host of CWD (Denker et al., 2024; Fig. 3).

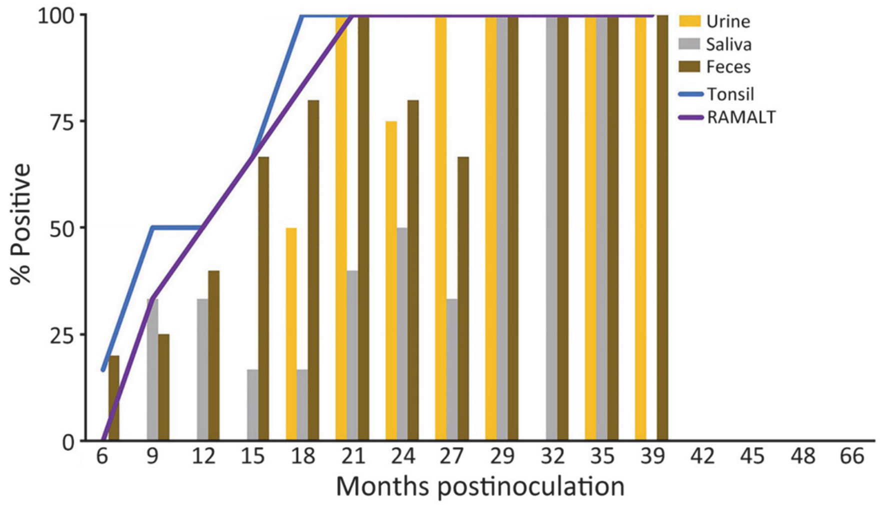

Figure 3. Percentage of prion-positive samples from white-tailed deer with chronic wasting disease that had the prion protein genotype 96GG in study of prion shedding in secreta, United States. Tissue samples and secreta were collected from deer after exposure to low oral doses of chronic wasting disease–positive brain tissue or saliva. RAMALT, recto-anal mucosa-associated lymphoid tissue.

Denkers et al., 2024

Minor issue 8

At some point in the manuscript, it would be beneficial to clarify the previously observed advantages of Ddi versus Mmo vaccination, or vice versa, to help readers understand the rationale for using both immunogens.

Both immunogens show efficacy, do their job. Ddi is a “self” antigen, Mmo more ‘foreign’ in this situation (host expressing cervid PrP). This is reflected by usually higher antibody titers for Mmo, but often better neutralization effects for Ddi sera (our previous publications). We are currently testing vaccination with both immunogens simultaneously (mixed) and using a chimeric immunogen expressing both mouse and deer PrP.

In a previous publication from our group, Abdelaziz at al., 2018, we used four different immunogens to test their protective effect against CWD in Tg-Elk mice. Both Ddi and Mmo significantly extended the survival of the CWD infected Tg-Elk mice. This led us to focus on these two immunogens in our subsequent studies to expand our knowledge regarding their effect on shedding.

Vaccination protects against CWD

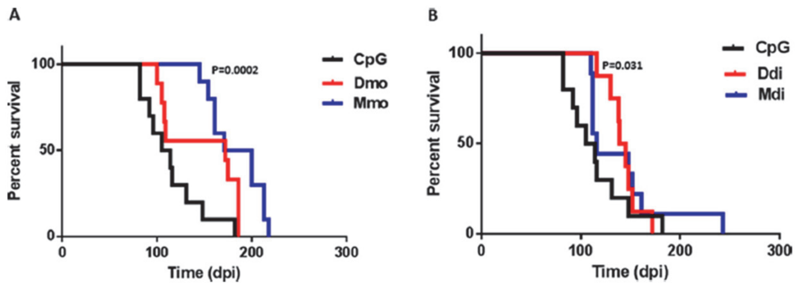

Figure 2. Immunization with monomeric or dimeric recombinant PrPs prolongs the survival in a CWD-infected TgElk mouse model. *A* and *B*, Kaplan-Meier survival curves of Dmo, Mmo, Ddi, and Mdi-vaccinated groups compared with control group (CpG-only) of female TgElk mice, inoculated via i.p. route with 1% BH of a terminally ill CWD-infected TgElk mouse. *A*, CpG control group, $n = 10$, median incubation time 109 days post-inoculation (*dpi*). Dmo-vaccinated group, $n = 9$, median incubation time 172 dpi ($p = 0.0509$; log rank test). Mmo-vaccinated group, $n = 10$, median incubation time 185 dpi ($p = 0.0002$; log rank test). *B*, CpG control group, $n = 10$, median incubation time 109 dpi. Ddi-vaccinated group, $n = 8$, median incubation time 142 dpi ($p = 0.031$; log rank test). Mdi-vaccinated group, $n = 10$, median incubation time 116 dpi ($p = 0.198$; log rank test).

Abdelaziz et al., 2018

Minor issue 9
The sample collection procedures, especially for urine, are not sufficiently detailed. Please specify:
- *Is urine collected individually or pooled during collection?*
- *What measures were taken to avoid contamination if animals were housed in the same cage?*
- *Were metabolic cages used to collect urine?*
- *Please also explain the details of how urine samples were processed.*

Clarifying statements were added to the Materials & Methods section (lines 470-474: "Regarding collection of urine, we did not use metabolic cages. However, we restrained the mouse in a tube and then placed the rear part of the body above a sterile petri dish and collected the urine using sterile tips to avoid any external contamination. The urine was collected individually and pooled before analysis because the volume obtained from each individual mouse was very minuscule.").

Minor issue 10
Panels d and f in Figure 1 are not explained in the figure legend. Please add appropriate descriptions.

Done as suggested.

Minor issue 11
In Figure 1, it does not make sense to discuss "delay" when the dpi timepoints at which samples were collected after euthanasia are different between animals. The PrPres signals are perfectly correlated with dpi. The statement in the figure legend "The delay in the neuro-invasion in the vaccinated groups represented in graph c and e" cannot be made unless all animals had been sacrificed at the same time.

We have deleted this statement. Unfortunately, our study did not contain scheduled euthanasia time points, this was more a result of unscheduled euthanasia due to intercurrent disease (mostly dermatitis and skin infections).

Minor issue 12

How do the authors explain that Ddi and Mmo induce so few common antibodies? It is surprising that, given the characteristics of Ddi and Mmo, specific antibodies for each antigen can be induced so distinctly. It is particularly surprising that antibodies induced with Ddi recognize the Mmo antigen so poorly. Please clarify this in the discussion.

We agree, it is surprising to see such pronounced differences. We think this is somewhat specific to the Elisa readout. As mentioned above (minor issue 8), Ddi is almost a true self antigen (2 aa different), Mmo has more “foreign” amino acids, in fact ~10% difference from deer PrP (21 residues). This makes Mmo more efficient in generating anti-PrP antibodies in hosts expressing a cervid PrP, some of which also detect deer PrP. On the other hand, we see cross-reactivity or cross-protection also when using Ddi as immunogen in such hosts. Both immunogens work *in vitro* (e.g. neutralization in cell culture or RT-QuIC; Abdelaziz et al., 2017) and *in vivo* (e.g. extension of incubation time; Abdelaziz et al., 2018). We tested sera from transgenic mice expressing cervid PrP vaccinated with Ddi in immunoblot and found some cross-reactivity for mouse PrP (lane 5 of attached blot; taken from Abdelaziz, 2018; Fig. 4), although cross-reactivity was better in the inverse situation.

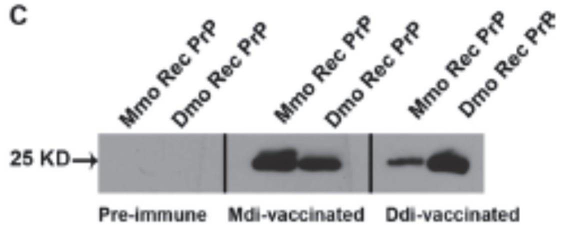

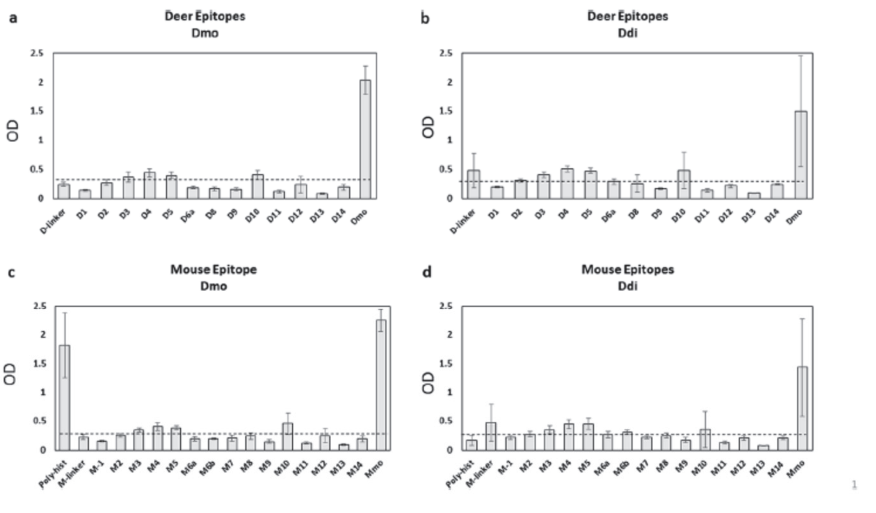

Figure 8. Linear epitope mapping for Dmo and Ddi post-immune sera. The optical density (OD) at 405 nm indicates the reactivity of sera from either Dmo (**a**,**c**) or Ddi (**b**,**d**) immunized mice against linear epitopes of deer (**a**,**b**) or mouse PrP (**c**,**d**) in ELISA. The dashed horizontal line represents the cut-off, which is 3 times the average of pre-immune sera. Data are presented as mean ± SD of results from three individual mice of each group. The amino acid sequences for linear epitopes is shown in Supplementary Tables 1 and 2.

In epitope mapping studies we found almost identical situations when using mouse or deer peptides for Ddi-vaccinated sera (taken from Abdelaziz et al., 2017; Fig. 8 b and d; 3 individual mouse sera were pooled). In the present manuscript, we only show cross-reactivity to deer peptides in SFig 1 (pooling 4 individual mouse sera), which again looks very similar for mice receiving Ddi or Mmo. We have clarified this aspect now better in the Discussion.

Minor issue 13
How does the 3-round PMCA + RT-QuIC approach compare to a potential alternative read-out consisting only of PMCA but with more rounds?

It was not better in our hands, less specific and more prone to false positives. There also is the risk of variability issues in serial PMCA that might affect the interpretation, as mentioned above by this referee as Major Concern 3.

Minor issue 14
Related to Major concern 2 regarding overstatement and the re-amplification read-out method: Given the way of quantifying by RT-QuIC following prior amplification, it does not make sense to distinguish between values of 50, 75, and 100. This could be misinterpreted as suggesting that results of 75, 50 versus 100, 100 represent different outcomes when, with this procedure, both should simply be considered positive. Since the authors have already explained what is considered positive and negative, they should standardize by indicating positive or negative. Otherwise, this misleads the reader by appearing to differentiate between values of 50, 75, and 100.

This comment refers to data shown in Fig. 3, where we have a pooling situation (fecal samples per cage). We think it makes sense to show it this way, to better address differences in vaccine efficacy on shedding. We resolve this issue better in Fig. 4 with samples from individual mice, where data indicate fewer positive ones in the vaccinated groups and also less seeding activity in the still positive samples.

Minor issue 15
In the urine study shown in Figure 5, were amplifications performed with additional rounds of PMCA beyond the three rounds used?

We did not perform PMCA with more rounds for urine.

Reviewer #3:
2. Since you have IPR data on serial dilutions of excreta, consider estimating reduction in SD50 by Reed-Meuncsh or Spearman-Karber methods to estimate titer reduction after vaccination, or provide a compelling argument why not.

We appreciate this comment which we had discussed internally for quite some time. We finally decided not to do so, given also the comments brought up by Referee 2 as Major Concern 2 (*Throughout the manuscript, the authors make statements that could be misinterpreted by readers regarding prion concentrations and fold-reductions…*). The values we present are the result of prior seed amplification (PMCA) and subsequent prion conversion (RT-QuIC), combining two highly sensitive assays that likely enlarge very small differences in initial PrP^{Sc} quantity upon amplification. We have addressed this in lines 411-414 of the Discussion: "…our IPR readout method combines purification and amplification by PMCA with a re-amplification by RT-QuIC…could mean that very small differences in initial PrP^{Sc} quantities could be enormously amplified in a non-linear way.").

3. Consider discussing that, despite the mice living longer, they still shed less prions early in disease course and may shed little or no additional prions during the additional thirty days that they live with CWD.

We have done this, lines 271-273 in Discussion.

4. GI systems of mice and cervids are very different. Briefly discuss this caveat when translating these results to the natural host.

Many thanks for this comment. We have added at lines 377-388: "One caveat of our vaccine study is that we vaccinated systemically. Wildlife vaccination most likely will need an oral vaccination approach, due to feasibility issues and for generating mucosal immunity that likely is needed to protect against oral CWD infection. Using mouse models for validating oral vaccination and for translating results to the natural host will be complicated by the fact that the GI systems of mice and cervids are very different. Compared with rodents, the ungulate stomach milieu comprises four compartments that feature different retention and passage times and are associated with varying pH conditions. Delivering vaccines orally in ruminants faces therefore significant challenges, including stability and degradation, limited mucosal permeability, and inefficient uptake by antigen-presenting cells. We have addressed this by using poly lactic co-glycolic acid (PLGA) nanoparticles that co-encapsulate vaccine and adjuvant (67). Studies in mice showed that this approach can induce both mucosal and systemic immune responses against PrP^C, studies in cervids are ongoing.".

5. Figure 1: Indicate in the legend "PrPRes levels in c and d, Brain, and e and f, Spinal cord" and define "SB".

Done as suggested.

6. Figure 5: Panels d and e hard to read

This has been corrected now.

7. Update the last column in SF7 from "Titer" to "Reciprocal Titer" (suggested) or "Fold-Dilution"

As suggested, was changed to fold dilution.
